# A Pilot Digital Intervention Targeting Loneliness in Youth Mental Health

**DOI:** 10.3389/fpsyt.2019.00604

**Published:** 2019-08-23

**Authors:** Michelle H. Lim, Thomas L. Rodebaugh, Robert Eres, Katrina M. Long, David L. Penn, John F. M. Gleeson

**Affiliations:** ^1^Centre for Mental Health, Swinburne University of Technology, Melbourne, VIC, Australia; ^2^Iverson Health Innovation Research Institute, Swinburne University of Technology, Melbourne, VIC, Australia; ^3^Department of Psychological and Brain Sciences, Washington University St. Louis, Missouri, MO, United States; ^4^School of Behavioural and Health Sciences, Australian Catholic University, Melbourne, VIC, Australia; ^5^Department of Psychology and Neurosciences, University of North Carolina Chapel Hill, North Carolina, NC, United States

**Keywords:** loneliness, social anxiety disorder, positive psychology intervention, digital intervention, youth mental health

## Abstract

**Background:** Loneliness is an emerging issue for young people, but yet many interventions to address loneliness in this group focus on providing social opportunities. While these sorts of interventions may appear to increase social connections, loneliness is more related to quality rather than quantity of social relationships. Thus, interventions addressing loneliness should focus on maximizing the quality of current relationships. Together with youth consumers both with mental ill health and those without, we developed a digital smartphone application (app) called +Connect. The 6-week program delivers positive psychology content designed to improve relationship quality. We tested the acceptability, feasibility, and safety of the program in lonely young people with or without a mental health diagnosis of social anxiety disorder. We used a mixed method study design to triangulate pilot quantitative and qualitative data in young people with and without social anxiety disorder (SAD).

**Method:** Nine participants with a diagnosis of social anxiety disorder (*M*_age_ = 21.00; *SD* = 1.41) and 11 participants with no mental health conditions (*M*_age_ = 20.36; *SD* = 2.16) completed the +Connect digital intervention.

**Results:** Those with social anxiety disorder reported less acceptable ratings on outcomes. Feasibility ratings, measured by uptake and app completion, met *a priori* threshold criteria in both groups. Those with social anxiety disorder yielded more attrition, with almost double the attrition rate compared with those without the disorder. There were no safety issues elicited during the pilot study. In terms of outcomes, exploratory analyses indicated that the app itself is likely to be beneficial rather than cause harm. Our qualitative data indicated both groups reported no negative outcomes and noted that positive outcomes were driven by three processes: reflection, learning, and real-life application. Further exploratory data on usability indicated room for improvement in terms of giving more support for different components of the app (i.e., challenges).

**Conclusion:** The pilot findings of this proof-of-concept app indicates some promise in terms of a second iterative version of +Connect.

## Introduction

Loneliness is a subjective experience of social isolation (1), and although such aversive feelings occur across the lifespan (2), young adults (18–29), together with older adults (65–79) are the most vulnerable to loneliness, reporting the highest prevalence compared to other age groups (3). Forming meaningful relationships with others is critical to our survival, and the lethality of loneliness is well established. Future poor health and increased risk of death is predicted by loneliness (4, 5). In addition, deleterious effects have been observed on various physical health conditions, including decreased immunity (6), increased inflammatory response (7), elevated blood pressure (8), decreased cognitive health (9), and faster progress of Alzheimer’s disease (10), to name a few.

The challenge of loneliness is magnified by its effects on mental health in addition to its effects on physical health. Loneliness is well known to be associated with increased mental health symptoms in both clinical (11, 12) and nonclinical populations (13–15). In longitudinal studies that examined older adults aged 50–68, loneliness predicted depressive symptomatology over and above potential confounding variables (e.g., demographics, stress, and social support) (16). Similarly, in a large-scale general population study of people aged 18 to 87 years old (*N* = 1,010), loneliness predicted more severe social anxiety, depression, and paranoia across a 6-month period (17). Given these results, targeting loneliness has the potential to prevent the development of more severe psychiatric symptoms.

Focusing interventions on youth might have the largest impact because loneliness and psychiatric symptoms are common in this age group and because of the potential for secondary prevention. In a large Danish study of adolescents and young people aged between 16 and 29 years old (*n* = 5,324), being female, having low levels of education, and living in a deprived area were risk factors for loneliness (18). Young people aged 12 to 25 are also at high risk of developing emerging mental illnesses (19). In the 2007 Australian National Survey of Mental Health and Wellbeing, young people aged 16–24 years have the highest prevalence of mental disorders, but the lowest rate of receiving services in the past 12 months (20, 21). Young adulthood is further marked with significant life changes from leaving school or home, to facing new social challenges such as higher education or work (22). Such social transitions mean that young adults may be more reliant than ever on their social networks for support (23). Having high-quality relationships can improve the young adult’s ability to adjust to new social environments (24, 25), buffering the effects of loneliness (26).

Interventions aimed at fostering social inclusion and enhancing social relationships can promote psychological well-being or promote recovery from problematic mental health symptoms (27). However, because loneliness is more related to the quality of social relationships than to the quantity (28, 29), interventions that focus solely on providing more social opportunities to the “lonely” individual have shown minimal benefit (30). This is because loneliness is not the same as being alone or physically isolated and is not strongly correlated with time spent alone (31). Hence, simply asking a “lonely” individual to join a group or interact with others provides either transient or minimal relief from loneliness. Cognitive models of loneliness elaborate on the distinction between loneliness and being objectively alone. Proponents of such models contend that while the adverse nature of loneliness motivates “lonely” individuals to connect with others, they are also hypervigilant to social threats, causing them to find evidence that people are not trustworthy or accepting (32, 33). In response, the “lonely” individual shows less prosocial behaviors toward others in an attempt to protect him or herself from rejection thereby eliciting rejection from others (32, 33).

The cycle outlined above suggests that a key target in reducing loneliness is to maximize the experience of social connection within *existing* relationships by helping the “lonely” individual to show more prosocial behaviors toward others. A positive psychology-based intervention provides a useful framework when addressing loneliness as it is designed to increase the meaningfulness of existing relationships, promote positive emotions, and focus on thriving during adversity (34–36). Positive psychology is the scientific study about what is right about the individual (as opposed to what is wrong), and it involves identifying positive characteristics, strengths, and psychological assets, which are inherent in a person irrespective of societal status (37).

Young people are well known to use digital tools extensively to connect with others, but existing social media apps may favor a large number of brief social interactions, as opposed to fewer and more meaningful relationships or designed to increase social support (38). Existing social media platforms carry a risk of alienation and cyberbullying (39, 40), which might contribute to more loneliness (41). This situation suggests the possibility of using a more positive app to build on strengths and reduce loneliness. This possibility comports well with the increase in digital platforms to either augment face-to-face mental health interventions or to simply engage young people who would otherwise not engage in mental health intervention (42). Although a digital health intervention that can deliver evidence-based health information is highly valuable and preferred by young people (43, 44), significant resources for development and testing are required.

We developed a proof-of-concept digital tool to address loneliness in young people. +Connect is a 6-week smartphone intervention using a strengths-based positive psychology framework. It is gamified and engaging. It delivers videos and posts daily in an attempt to convey evidence-based concepts known to strengthen relationships and increase social connections. Over 2015–2017, young people aged 18 to 25 participated in a series of focus groups. The groups included members that ranged from young people with no mental ill health, to those with a history of high prevalence disorders (e.g., depression, social anxiety) to those with psychotic disorders. Feedback from initial focus groups also recommended that content should be delivered in short but frequent bursts (as opposed to longer and dedicated time) (45). We opted to develop a smartphone app over other digital platforms because of its mobility and accessibility (46–48). The group then provided feedback on design (i.e., fonts, colors, layout), functionality (e.g., task completion and gamification), and language (e.g., written task and video content).

In a pilot study, we employed a mixed methods approach using both quantitative and qualitative data in order to deepen our understanding of how young people experience +Connect. The primary study aim was to examine the acceptability, feasibility, and safety of +Connect in young people with or without social anxiety disorder. First, we anticipated that +Connect would be acceptable to both groups of young people. This would be demonstrated by participants reporting higher than *somewhat* in their satisfactory and helpfulness ratings across a series of criteria, including understanding, enjoyment, and content helpfulness.

Second, we anticipated that +Connect would be feasible across three key factors: uptake, attrition, and app completion. For uptake, we anticipated that at least 50% of people who were interested would attend a baseline assessment. For attrition, we anticipated no more than 30% attrition rate for both groups. We further considered a participant to be a drop out if he or she ceased using the app for >3 consecutive days, and the researchers were unable to contact the participant. For app completion, we hypothesized that participants would complete at least 70% of the program (equivalent to 30 of the 42 days of content). We explored other possible feasibility factors *via* the qualitative interview. Third, we anticipated that +Connect would be safe to use for lonely young people and to assess safety, we measured the number of adverse events that occurred during the course of the study.

Fourth, in an exploratory analysis, in order to determine if the intervention reduces loneliness severity, we estimated the plausible effect size of +Connect on loneliness ratings *via* a latent trajectory model in an exploratory analysis. We included measures of mental health such as social anxiety and depression symptom severity that could influence loneliness severity. Last, in a second exploratory analysis, we measured the usability of the app (e.g., functionality, navigation) and acceptability ratings around the app design, concepts, and delivery components.

## Materials and Methods

### +Connect Application Information

We opted to relay socially oriented content *via* video material wherever possible. We developed three types of videos: 1) shared experience videos (SEVs) using young people with lived experiences of loneliness (49); 2) expert videos (EVs) featuring academics (50), or 3) actor^1^ videos (AVs) demonstrating concepts *via* modeling (51), all of which have already been utilized in previous digital interventions.

Key concepts relayed *via* a smartphone app means that content had to be concise and frequent as opposed to other internet delivered interventions, which may require longer but more infrequent discrete blocks. +Connect’s program was designed to be delivered in less than 5 min over 42 days (6 weeks). Participants are shown a home screen when they opened the app. They were asked to log their mood states using a mood evaluation tracker. They are then directed to a task which was delivered either: 1) *via* text and images (e.g., an Instagram format); 2) SEVs featuring young people with lived experiences; 3) EVs featuring academics introducing core concepts; or 4) AVs featuring actors who would model interactions within specific social contexts.

Videos were designed to be brief (i.e., 1.21 to 4.38 min). AV scripts were written by a scriptwriter (under 25 years of age) and reviewed in a series of focus groups with young people (students, those with a history of high prevalence and serious mental illness). Two coders unrelated to this study (graduate students under clinical training) rated the content of each SEV on whether it achieved the aims of the modules (e.g., gratitude video: to relay that expressing gratitude can feel awkward at first, and it is more than saying thank you). These processes meant that material was checked and refined to ensure that concepts were simple to understand, youth friendly, and relatable. Participants completed daily tasks and were asked to answer a series questions related to the post or video, using a multiple choice or True/False format. Daily tasks did not exceed past 5 min. The gamification processes include winning points and badges through task completion, mood monitoring logins, progress through the app, and winning challenges (52–54). **Supplementary Table 1** outlines what was delivered within +Connect app. The content was developed by ML, JG, TR, and DP. There are different tasks (e.g., the gratitude exercise, showing gratitude) that can sit under one general module (e.g., Gratitude).

### Participants

Twenty participants aged 18 to 23 years old were recruited for the study. Nine participants with social anxiety disorder (*M* = 21.00, *SD* = 1.41) were recruited from a local youth health service, while 11 participants with no diagnosable mental health disorder (*M* = 20.36, *SD* = 0.52) were recruited through convenience sampling from an Australian university. See **Table 1** for more participant demographic information.

**Table 1:** Demographics of participants across groups.

Demographic Variable *M(SD) or %*	SAD	Students
% Female	44.44%	45.45%
Age	21.00 (1.41)	20.36 (2.16)
Ethnicity Asian Australian or Asian White (including Caucasian, European, Australian) African Australian Multi-Racial Other	22.22% 78.78% 0% 0% 0%	36.36% 45.45% 0% 9.09% 9.09%
Relationship status (% Single)	89.89%	81.81%
Living status Living alone Residing with housemates Residing at home with immediate family Residing with relatives Residing in college	11.11% 33.33% 44.44% 0.00% 11.11%	0.00% 54.54% 36.36% 0.00% 9.09%
If residing with others, number of people in household One other person Two other people Three other people Four other people Five or more other people	12.50% 25.00% 37.50% 0.00% 25.00%	27.27% 0% 36.36% 18.18% 18.18%
Completed education (in years)	13.67 (1.50)	14.00 (2.24)

SAD refers to social anxiety disorder.

### Materials

Participants attended three assessments: Time 1 (T1), baseline; Time 2 (T2), post-treatment (after completing at least 33 days of +Connect); and Time 3 (T3), 3-month follow up (conducted 3 months after the T2 assessment). All measures were administered at all timepoints except for a) the Structured Clinical Interview for Diagnostic and Statistical Manual of Mental Disorders (SCID-5-RV; 55), which was only administered at baseline, and b) the qualitative interview, which was conducted only at T2.

#### The Structured Clinical Interview for Diagnostic and Statistical Manual of Mental Disorders (SCID-5-RV; 55)

The SCID-5-RV depression and social anxiety modules were administered at baseline to determine the study eligibility and clinical diagnosis for the SAD group. Thirty percent amount of the assessments was randomly selected and independently rated by another coder with 100% agreement on diagnosis.

#### The Revised UCLA Loneliness Scale (UCLA-LS; 34)

The UCLA-LS, a 20-item self-report scale, was used as a measure of loneliness severity. It uses a 1 (Never) to 4 (Always) Likert-type scale. The measure consists of both positively and negatively worded items that assess loneliness (e.g., How often do you feel that you are no longer close to anyone)? The UCLA-LS has been shown to correlate negatively with life satisfaction and perceived social support, thus supporting its convergent validity with related constructs (56). Internal consistencies αs ranged from 0.90 to 0.95 across time.

#### The Social Interaction Anxiety Scale (SIAS; 56)

The original SIAS is a 20-item self-report questionnaire that measures anxiety-related reactions to different social interactions (e.g., *I get nervous if I have to speak with someone in authority*). The 17 *straightforwardly-worded* items (S-SIAS) were found to be more valid indicators of social interaction anxiety than the reverse-scored items across different samples (57). For this reason, we used the straightforward items. Internal consistencies ranged between αs = 0.91 and 0.94 across time.

#### Centre for Epidemiological Studies – Depression (CES-D; 58)

The CES-D is a 20-self-report measure of depressive symptoms, which employs a 0 (rare or none of the time) to 3 (most or all of the time) Likert-type scale. Scores are summed to create a total score indicative of depression symptomatology, where higher scores indicate the presence of more symptomatology. The CES-D has strong internal reliability (58). Internal consistencies for the CES-D ranged from αs = 0.88 to 0.90 across time.

#### Semi-Structured Qualitative Interview

Participants were invited to complete a semi-structured interview regarding their experiences using +Connect at T2. The questions are provided in **Table 2**. The interview was transcribed verbatim prior to analysis.

**Table 2:** Study inclusion and exclusion criteria for social anxiety disorder and student groups.

Inclusion Criteria	Exclusion Criteria
1. Aged 18–25 years^a^ 2. Engaged with a current mental health service, general practitioner (or was engaged at time of assessment)^a^^,^^b^ 3. Provided consent to contact current/previous mental health worker or general practitioner should risk issues arise^a^^,^^b^ 4. Own a smartphone (Android or iOS)^a^ 5. Identified a desire to connect with others^a^ 6. Current DSM V of SAD assessed by the SCID-5^b^^,^^c^ 7. UCLA Loneliness Scale score >38^d^	1. Presence of moderate or severe risk issues, i.e., deliberate self-harm and suicidality in the past month^a^ 2. Psychiatric hospitalization in the past month^a^ 3. Substance abuse or dependence in the past month^a^ 4. Known diagnosis of an Axis II personality disorder^a^

a Items checked at the initial telephone screen.

b Only applicable to those with social anxiety disorder.

c Students were accepted only if they did not meet criteria for social anxiety disorder as assessed by the SCID-V.

d There is no known threshold for problematic or severe loneliness, but a score of 38 and above was used to indicate above the median score across different samples (Russell, 1996).

### Design and Procedures

Human research ethics approval was obtained from the university ethics board, and written informed consent was obtained from participants. We recruited young people who were assessed to be lonely. There were two groups: 1) young people with social anxiety disorder and young people with no current mental health disorder. Participants with social anxiety disorder were recruited *via* their case manager at the local youth mental health service. A student group was recruited *via* print media placed around the local university. All potential participants were first screened *via* telephone to assess their eligibility for the study. See **Table 3** for inclusion–exclusion criteria for each group.

**Table 3:** Semi-structured interview script.

Question Type	Question
Experience of the app	How did you feel after finishing +Connect?
	What was it like to go through all the 42 days?
	What did you think about the different types of videos (Probe: SEV vs AV vs Expert)?
	What was it like doing the challenges (Probe: applying +Connect to daily life)?
	What would have encouraged you to do the challenges more?
	What was it like to do all the tasks? (Probe: What could have encouraged you to complete them)?
Benefits and challenges of the app	What part of +Connect was the most helpful, and why?
	What part of +Connect did you find the most fun, and why?
	What did you find challenging about +Connect and why?
	What was it like to focus on your strengths and positive things?
Functionality of the app	How did using the app fit in with your daily life?
	Where and what time did you usually use the app?
	Research tells us that people like apps to be interactive – are there any interactive features that you would have liked to have seen in the app?
	Were all the sections in +Connect relevant to you? Why or why not?
	Finally, do you have suggestions that can help us improve the app for other people that use it?

The research assistant administered the UCLA Loneliness three-item scale (59) over the telephone; those who scored 5 or more and did not meet the exclusion criteria were invited to a face-to-face baseline assessment, during which they completed the remaining measures. At the baseline assessment, participants provided consent to the study and complete the UCLA-LS. The research assistant scored the scale and proceeded with the SCID-5-RV if the score is above 38. The SCID-5 was audio recorded in order to conduct inter-rater reliability on the clinical diagnosis. Participants were excluded at this point if they met any of the exclusion criteria. Participants who were assessed to be eligible then completed the remaining assessments.

Once participants completed the baseline assessment, research assistants introduced and oriented the participant to the app (i.e., completing the first day with the participant). The participant was briefed on the purpose, design, and functionality of the app, and shown how to navigate the different components. Researchers were able to monitor the progress of the participants *via* a webpage. Participants were contacted weekly for brief check in (either *via* text or a phone call). This was conducted to ensure technical issues were reported swiftly and to identify and address emerging risk issues during the course of the study. Participants were reimbursed for the completion of each assessment ($15/h). If they completed every day of the app, they were reimbursed up to $1.90 per day. This was done to offset the cost of data use outside WIFI zones.

### Data Analytic Plan

A mixed methods approach included both descriptive statistics and qualitative analysis. Specifically, participant interviews and qualitative survey responses were analyzed using content and thematic analysis. These data were used to support quantitative data. An exploratory analysis using a latent trajectory model of the UCLA-LS was also used to estimate the effects of the intervention on young people. Bayesian estimation was used in Mplus (60) to provide a credible interval for the intervention’s effect over time, assuming a linear slope across reporting periods. We consider these analyses exploratory because they were clearly underpowered to detect anything but a large effect size given the sample size. We also reported Cohen’s *d* for effect size. Consistent with all pilot interventions, acceptability, feasibility, and safety are the primary outcomes. *Acceptability* was assessed at post-intervention using satisfaction ratings on questionnaires, content helpfulness ratings, and qualitative interview data that support these measures. Feasibility was assessed by considering four key factors: uptake, attrition, retention, app completion, and *engagement*. *Uptake* was defined as the number of potentially eligible young people who attended the baseline assessment. *Attrition* was defined as the number of participants who attended the baseline assessment but failed to log into the app for more than three consecutive days and where researchers were unable to contact the participant. *App completion* was defined as accessing and completing at least 70% of the app (30 out of 42 days). Consistent with most pilot interventions and recommendations (61), we also measured *Safety*, which was operationalized as the incidence of serious adverse events (e.g., hospitalization and self-harm) during the course of the study (62).

## Results

### Acceptability

#### App Satisfaction Ratings

We set *a priori* threshold of outcome satisfaction ratings of more than 70% of participants in each group would rate higher than *somewhat satisfied* in their satisfactory ratings (that ranged from *very satisfied*, *somewhat satisfied*, *not at all satisfied*) across a set of criteria relating to different outcome ratings regarding ease of understanding, enjoyment in life, and content helpfulness. **Student app satisfaction ratings.** Around 72.73% of participants in the student group said that they were somewhat or very satisfied with each outcome criterion (see **Table 4**). Furthermore, all participants found +Connect easy to understand; however, 18.18% to 27.27% reported being not at all satisfied with several components of +Connect, including increasing social confidence and creating new relationships. See **Table 4** for details of the outcome satisfaction ratings. **Social anxiety group app satisfaction ratings.** As shown in **Table 5**, at least 50% of participants in the social anxiety group rated themselves as either somewhat or very satisfied with each of the outcome criterions. Similar to students, all of the participants with SAD reported that +Connect was easy to understand and helped them accept their mental health symptoms. However, 25.00% to 50.00% reported being not at all satisfied with several components of the app, including creating new relationships and increasing social confidence.

**Table 4:** Post-intervention outcome satisfaction ratings of the +Connect for the student group.

Question	Very satisfied		Somewhat		Not at all satisfied	
	*n*	%	*n*	%	*n*	%
Ease of understanding	9	81.81%	2	18.18%	0	0%
Look forward being with people	4	36.36%	5	45.45%	2	18.18%
+Connect helped me enjoy life	6	54.54%	3	27.27%	2	18.18%
+Connect helped me feel connected with others	2	18.18%	7	63.63%	2	18.18%
+Connect helped increase social confidence	5	45.45%	3	27.27%	3	27.27%
Helped create new relationships	3	27.27%	5	45.45%	3	27.27%
Helped accept mental health symptoms	5	45.45%	4	36.36%	2	18.18%

**Table 5:** Post-intervention outcome ratings of the +Connect for the SAD group.

Question	Very satisfied		Somewhat		Not at all satisfied	
	*n*	%	*n*	%	*n*	%
Ease of understanding	7	87.50%	1	12.50%	0	0%
Look forward being with people	1	12.50%	5	62.50%	2	25.00%
+Connect helped me enjoy life	1	12.50%	5	62.50%	2	25.00%
+Connect helped me feel connected with others	3	37.50%	3	37.50%	2	25.00%
+Connect helped increase social confidence	1	12.50%	5	62.50%	2	25.00%
Helped create new relationships	1	12.50%	3	37.50%	4	50.00%
Helped accept mental health symptoms	5	62.50%	3	37.50%	0	0%

SAD refers to Social Anxiety Disorder.

#### App Content Helpfulness

Additional acceptability questions related to how helpful the content was. **Student group content helpfulness.** Overall, students tended to find the modules of +Connect helpful, with 27.27% to 81.81% reporting the modules to be either *helpful* or *very helpful*. If we extend positive ratings to include *somewhat helpful*, we see that 91.01% to 100% of the participants reported +Connect to be helpful. In interviews, student participants reported Three Good Things as their favorite module (*n* = 7), e.g., *“I found the ones that were about how to make you feel better about yourself. So ones like three good things, for example….those bits I found most helpful.”* Those modules that students reported most as not relevant in their lives were self-disclosure (*n* = 2) and balanced relationships (*n* = 2). This was primarily because they felt they were “already aware of all that stuff” or had not experienced a situation in which the information would be relevant, i.e., *“Balanced relationships … I really didn’t relate to myself because I never had like that kind of relationship where it was always imbalanced or one-sided.”*

All students found the shared experience and expert videos *somewhat* or *very much* useful and enjoyable, while 81.81% of students found the actor videos were useful and 72.72% rating them as enjoyable (see **Supplementary Table 2** for details). This differed somewhat from the qualitative findings; while four students reported preferring the shared experience videos, one student found them boring and hard to relate to, *“Because when people actually shared their experiences, they are feeling that emotion, but you can’t feel that.”* Three students instead reported a preference for the actor videos. However, a further two student participants reported finding the actor videos *“kind of forced,” “kind of creepy,” and “fake.”* Three participants reported the expert videos as their favorite type of video.

**Social anxiety group content helpfulness.** Overall, participants with SAD tended to find the modules of +Connect helpful with 25% to 100% reporting modules to be either helpful or very helpful (see **Supplementary Table 3** for details). If we include somewhat helpful as a positive rating, we see that 75.00% to 100% of the participants with SAD reported +Connect to be helpful. In interviews, participants with SAD also reported Three Good Things as their favorite module (*n* = 3). Two participants with SAD reported that the savoring module was not relevant in their lives as *“It felt a bit wishy washy and I didn’t really know how to relate that too much of my life.”*

Participants with SAD reported a different pattern of preference for the video types in comparison to the student, in that 87.50% reported shared experience videos as being *somewhat* or *very much* useful, while 75.00% reported them being *somewhat* or *very much* enjoyable. This was supported, in part, by the qualitative results; five participants with SAD endorsed the shared experience videos as *“genuine,” “inspiring,”* and *“easy to relate to,”* while two participants with SAD felt that the content was sometimes *“fake”* or *“not relevant to [their] own experience.”* For the expert and actor videos, 75.00% of the participants with SAD rated the videos as *somewhat* or *very much* useful, while only 50.00% rated the expert videos as either *somewhat* or *very much* enjoyable and 62.50% rated the actor videos as *somewhat* or *very much* enjoyable. This difference in the rating of actor video usefulness and enjoyable was reflected in the qualitative findings. Four participants with SAD reported preferring the actor videos over the shared experience videos as *“you actually see how they are feeling and you understand how things are.”* However, two participants with SAD agreed with the student participants who found the actor videos *“a bit cheesy.”*

### Feasibility

#### Uptake

We set *a priori* threshold of at least 50% of people who were eligible would attend a baseline assessment. **Student uptake:** One hundred and one participants initially expressed interest in participating in the project over a 10-month period. Fifty-one students did not want to participate in the context of a research project but were given access outside the trial. Of the remaining 50 students, 22 were found to be potentially eligible after the initial phone screen; the remaining 28 were screened out primarily for not identifying as lonely or needing to connect, exceeding the age requirements, or having a mental illness. Of the 22 people who were potentially eligible for the study, we were unable to contact two participants for a baseline assessment, leaving only 20 participants who were invited to a baseline assessment. Hence, the uptake was around 90% with 2 out of 22 participants (around 91% uptake). **Social anxiety group uptake.** Nineteen young people were presented as potential participants from a local health service over a 12-month period. Two of these people were non-responsive to telephone attempts, while two people were ruled ineligible for the study during the phone screen for substance abuse (one participant) and increased risk to safety (one participant). The remaining 15 people were eligible to complete the baseline assessment, and of these, two people did not attend the baseline or did not finish the baseline assessment. The remaining 13 participants were enrolled in +Connect. Therefore, the uptake for social anxiety is around 13 out of 15 participants (around 87% uptake).

#### Attrition

We set *a priori* threshold of a 30% attrition rate for both groups. **Student group attrition.** There was a rate of 15.38% with two participants dropping out of the study citing no reason. **Social anxiety group attrition.** There was an attrition rate of 30.76% with 4 participants out of 13 dropping out of the study, and the only reason cited was time commitment.

#### App Completion

We set *a priori* app completion rate of 70% (30 out of 42 days) for both groups. **Student group app completion.** Participants completed 90.26% of the app (*M* = 37.91 days, *SD* = 5.09) exceeding the *a priori* requirement of 30 out of 42 days. **Social anxiety group app completion.** Participants (*n* = 9) completed approximately 84.66% of +Connect (*M* = 35.56 days, *SD* = 7.78), exceeding our *a priori* requirement of 30 days out of 42.

#### Other Feasibility Factors

There were additional feasibility factors relating to time burden, and difficulties with app components that were elicited. **Time burden.** Around 78.95% of the participants (15/19) found that the app did not create a significant time burden, reporting they used the app for 3 or more minutes per day, while the remaining 21.05% (4/19) reported using the app less than 3 min per day^2^. In interviews, one SAD and one student participant reported difficulties with the length of the strength challenge, and one SAD participant reported difficulties with the longer videos, i.e., *“When they started becoming four minutes and such, they got really really difficult to concentrate on them, or just like, find the time to actually do that.”* **Maintaining engagement.** The greatest difficulty participants with SAD reported during interviews was remembering to complete the challenges and tasks (*n* = 5), e.g., *“It was quite difficult to remember for me. Cos you know, even though like occasionally it would give me like a notification say I’ll forget to do it because I might have like gone to work or you know, I’ve got uni work I’m doing or whatever. I tried at one point to make it like a daily thing where I’d wake up and I’d do it. But that didn’t really work out. So it was hard to keep consistent.”* Conversely, only two student participants reported difficulties remembering to complete the app. It seemed that for student participants, it became part of their routine more easily, i.e., *“doing the app was just a daily task and then I think because there were some coming around it was just making interest to like continue on with the app.”* This may partly be due to more control participants (*n* = 4) reporting being motivated by the app gamification than clinical participants (*n* = 1). **Difficulties with app components.** Three participants with SAD also reported difficulties completing the challenges. This seemed to be primarily due to social anxiety severity levels, i.e., *“these kind of challenges I thought would take a lot of confidence in me. I just procrastinated on them a bit.”* Participants suggested breaking down the challenges into smaller and shorter components to make completion easier, i.e., *“maybe simple, easy challenges like ‘today, compliment a stranger’s outfit’ or something. I think if I did those kind of exercises each day, it will help me a lot in my social anxiety.”*

### Safety

No participant in either group reported any adverse event during the program.

### Health and Wellbeing Outcomes

First, we report the descriptive scores, means, and standard deviations across the two groups across time (see **Table 6** for descriptive statistics). Because our intent was to be descriptive, we do not provide tests of between-group differences, but we do describe changes over time within group. Next, we used a latent trajectory model of the UCLA-LS to estimate the effects of the intervention on young people. Bayesian estimation was used in Mplus (60) to provide a credible interval for the intervention’s effect over time, assuming a linear slope across reporting periods.

**Table 6:** Descriptive statistics for student and SAD groups across loneliness and secondary outcomes.

	Students (*n* = 11)			SAD (*n* = 9)		
Measure	Baseline *M(SD)*	Post-treatment *M(SD)*	3-month Follow-up *M(SD)*	Baseline *M(SD)*	Post-treatment *M(SD)*	3-month Follow-up *M(SD)*
UCLA-LS	48.18(7.85)	42.70(11.61)*	40.40(11.82)*	57.00(5.61)	51.67(6.89)	49.56(7.07)
S-SIAS	29.18(12.34)	21.64(14.00)	22.00(11.96)	43.22(7.56)	37.22(10.49)	34.89(13.80)
CES-D	11.55(7.10)	8.45(7.37)	8.45(6.93)	21.89(7.75)	14.00(5.51)	15.56 (8.88)

*n = 10 because one participant had missing data on item. SAD refers to Social Anxiety Disorder. UCLA-LS refers to University of California Loneliness Scale. S-SIAS refers to straightforward items from Social Anxiety Interaction Scale. CES-D refers to Centre for Epidemiological Studies Depression.

#### Student Group Descriptives

The UCLA-LS, S-SIAS, and CES-D scores decreased from baseline to post-intervention and 3 months post intervention.

#### Social Anxiety Group Descriptives

The UCLA-LS and S-SIAS scores decreased in a linear trend from baseline to 3 months post-intervention for the SAD group. However, CES-D scores decreased from baseline to post-treatment, but scores appeared to regress toward their baseline at the 3-month post-intervention period.

#### Combined Exploratory Quantitative Outcomes

Across the entire group of participants, loneliness showed a mean negative slope (*M* = −3.82, 95% Credible Interval [CI] −5.54 – −2.17). On the average, participants’ UCLA-LS scores decreased by 7.64 points (where the standard deviation at baseline was 8.11) by follow-up, suggesting a large effect (Cohen’s *d* = 0.94). If anything, results were stronger for students than the group overall (*M* = −4.38, 95% CI −8.45 – −0.66, *d* = 1.12, based on an *SD* of 7.84). The effect appeared similar in size, although it had a wider confidence interval for the group of participants with SAD (*M* = −3.39, 95% CI −7.61 – 0.41, *d* = 1.20, based on an *SD* of 5.61). In each analysis, the slope had a significant variance, indicating meaningful variation in how participants responded to the intervention.

#### Qualitative App Outcomes

In interviews, 75% of the participants reported at least one positive outcome from using the app. These primarily included increased positive affect (e.g., *“When I was using it more regularly … I felt a lot more happy with myself”*) and improved social interactions (e.g., *“I know more friends and can talk with them more.”*). Four participants (*n*_clin_ = 2, *n*_control_ = 2) reported no positive outcomes from using the app, (e.g.,* “it only takes up such a small part of your day that it’s not like it changed my lifestyle in any big dramatic way”*). No participants reported negative outcomes from using the app. Positive outcomes seemed to be driven by three main processes induced by the app: 1) reflection (*n*_clin_ = 5, *n*_control_ = 5); 2) learning (*n*_clin_ = 6, *n*_control_ = 8); and 3) real-life application (*n*_clin_ = 4, *n*_control_ = 5).

##### Reflection

This factor seemed to be the primary process underlying the increase in positive affect reported by participants, e.g., *“I don’t really tend to reflect on stuff that much and it helped me to go ‘oh, I could do that, and it would be productive’.”* Participants reported using the challenges, video content, and mood logs as ways of stimulating reflection.

##### Learning

In terms of learning processes, while videos were the main source of psychoeducational information, several participants (*n*_clin_ = 4, *n*_control_ = 3) highlighted the key role of the after-video questions in their learning process, i.e., *“The questions were definitely good. They were definitely smart because they made you, made you watch the video. You wanted to get them right because you learnt something.”*

##### Real Life Application

While some participants (*n*_clin_ = 2, *n*_control_ = 2) found that the app provided little new knowledge, they nonetheless valued how it provided revision of existing or commonsense knowledge, e.g., *“this stuff you just rarely notice, but it’s just a little bit reminder that you should be doing this to people around you.”* This revision often led participants to attempt to apply the lessons in real life, either informally, or more often, through the challenges: *“the challenges … just meant that it became a lot more … like oh ‘I can actually take it out into the world and do some of the things it suggested’.”* Participants reported finding this real-life application both rewarding and challenging, i.e., *“I feel like I’ve, there was a lot of information that at first seemed like common sense, to me, but although it may seem like common sense, it’s actually like important that we know those things because once we actually implement it, it actually makes a difference in our lives,” “Challenging? Trying to put it into real life context. And actually do it.”*

Three clinical participants reported an intent to apply +Connect skills in future social interactions, primarily due to a lack of current opportunities to apply the skills, e.g., *“because I don’t really have any friends or relationships [the app is] not relevant to me right now … But I feel like if I got a relationship it would be helpful. I definitely learned a lot, and I did take quite a bit of stuff from it.”*

### Usability

We measured variables related to functionality, design, and delivery of concepts in order to better design a second iterative version of +Connect. **Student group usability.** Overall, students found +Connect to be a usable smartphone application with 72.72% to 100% of participants rating that they *agree* or *extremely agree* that +Connect was easy to navigate, the format made sense, and that the language was easy to understand. Similarly, 72.72% to 100% rated that they *agree* or *extremely agree* that they liked the color scheme, fonts, photos, and videos. This was only partially supported by student participants’ qualitative feedback, with the majority of suggestions for app improvement relation to app navigation and design (*n* = 8)^3^. Student participants also reported finding the app questions were too easy (*n* = 6) and reporting being able to answer them correctly without watching the videos. They suggested increasing the difficulty of the questions in future versions. Around 27% of students did not enjoy challenges within the app, indicating that some work is required to improve the challenges (see **Table 7** for more details). **Social anxiety group usability.** Participants with SAD found +Connect to be a usable smartphone application as indicated by 100% of participants rating that they *agree* or *extremely agree* that +Connect was easy to navigate, the format made sense, and that the language was easy to understand. Furthermore, 62.50% to 87.50% of the participants rated that they *agree* or *extremely agree* that they liked the color scheme, fonts, photos, and videos. The qualitative feedback of the participants with SAD mirrored that of the student participants in conflicting with the quantitative results; five participants with SAD suggested improvements in app navigation and design, and five reported that the app questions were too easy. Similar to students, those with SAD did not find challenges particularly enjoyable. Furthermore, almost 50% of those with SAD reported that they did not find the badge reward scheduling system encourage participation, suggesting that a different reward schedule either by item or reward schedules will need to be revised (see **Table 8** for more details).

**Table 7:** Ratings related to functionality, design, and delivery of concepts for the student group.

Question	Extremely Disagree		Disagree		Neutral		Agree		Extremely Agree	
	*n*	%	*n*	%	*n*	%	*n*	%	*n*	%
**Acceptability**										
Enjoyed using +Connect	0	0%	0	0%	2	18.18%	6	54.55%	3	27.27%
+Connect was useful	0	0%	0	0%	2	18.18%	5	45.45%	4	36.36%
Enjoyed content	0	0%	0	0%	2	18.18%	5	45.45%	4	36.36%
Understand the ideas	0	0%	0	0%	1	9.09%	2	18.18%	8	72.73%
Gained a lot	0	0%	0	0%	3	27.27%	6	54.55%	2	18.18%
Could relate to content	0	0%	0	0%	1	9.09%	5	45.45%	5	45.45%
Relate to characters	0	0%	0	0%	0	0%	9	81.82%	2	18.18%
Videos helped with content	0	0%	0	0%	0	0%	7	63.64%	4	36.37%
Videos were entertaining	0	0%	0	0%	2	18.18%	6	54.55%	3	27.27%
Questions helped with content	0	0%	0	0%	2	18.18%	6	54.55%	3	27.27%
Questions were the right level of difficulty	1	9.09%	0	0%	1	9.09%	4	36.37%	5	45.45%
Enjoyed challenges	1	9.09%	2	18.18%	0	0%	4	36.37%	4	36.37%
Badges encouraged participation	1	9.09%	0	0%	3	27.27%	3	27.27%	4	36.36%
**Usability**										
Easy to Navigate	–	–	1	9.09%	0	0%	7	63.63%	3	27.27%
Format made sense	–	–	1	9.09%	1	9.09%	6	54.54%	3	27.27%
Language is easy to understand	–	–	–	–	0	0%	6	54.54%	5	45.45%
Liked color scheme	–	–	–	–	3	27.27%	5	45.45%	3	27.27%
Liked Fonts	–	–	–	–	3	27.27%	6	54.54%	2	18.18%
Liked Photos	–	–	–	–	3	27.27%	6	54.54%	2	18.18%
Content is interesting	–	–	–	–	3	27.27%	5	45.45%	3	27.27%
Liked Videos	–	–	–	–	0	0%	10	90.91%	1	9.09%

**Table 8:** Ratings related to functionality, design, and delivery of concepts for the SAD group.

Question	Extremely Disagree		Disagree		Neutral		Agree		Extremely Agree	
	*n*	%	*n*	%	*n*	%	*n*	%	*n*	%
Acceptability										
Enjoyed using +Connect	0	0%	0	0%	2	25.00%	5	62.50%	1	12.50%
+Connect was useful	0	0%	0	0%	0	0%	6	75.00%	2	25.00%
Enjoyed content	0	0%	0	0%	1	12.50%	5	62.50%	2	25.00%
Understand the ideas	0	0%	0	0%	1	12.50%	3	37.50%	4	50.00%
Gained a lot^a^	0	0%	1	12.50%	1	12.50%	5	62.50%	0	0%
Could relate to content	0	0%	0	0%	0	0%	8	100.00%	0	0%
Relate to characters	0	0%	0	0%	0	0%	6	75.00%	2	25.00%
Videos helped with content	0	0%	0	0%	0	0%	6	75.00%	2	25.00%
Videos were entertaining	0	0%	2	25.00%	2	25.00%	3	37.50%	1	12.50%
Questions helped with content	0	0%	0	0%	0	0%	7	87.50%	1	12.50%
Questions were the right level of difficulty	1	12.50%	1	12.50%	1	12.50	3	37.50%	2	25.00%
Enjoyed challenges	0	0%	1	12.50%	4	50.00	3	37.50%	0	0%
Badges encouraged participation	3	37.50%	1	12.50%	0	0%	4	50.00%	0	0%
Usability										
Easy to navigate	0	0%	0	0%	0	0%	4	50.00%	4	50.00%
Format made sense	0	0%	0	0%	0	0%	5	62.50%	3	37.50%
Language is easy to understand	0	0%	0	0%	0	0%	3	37.50%	5	62.50%
Liked color scheme	0	0%	0	0%	2	25.00%	5	62.50%	1	12.50%
Liked fonts	0	0%	1	12.50%	2	25.00%	2	25.00%	3	37.50%
Liked photos	0	0%	0	0%	1	12.50%	5	62.50%	2	25.00%
Content is interesting	0	0%	0	0%	2	25.00%	4	50.00%	2	25.00%
Liked videos	0	0%	0	0%	1	12.50%	4	50.00%	3	37.50%

*SAD refers to Social Anxiety Disorder.*

## Discussion

### Key Findings

+Connect is a digital intervention designed to target loneliness in young people. Because loneliness is more related to the quality of relationships as opposed to quantity, we employed a positive psychology approach to help young people identify their strengths, increase their positive affect, and focus on building the intimacy within existing relationships. We piloted the tool in both lonely young people with and without social anxiety disorder.

Overall, we found higher acceptability ratings across different ratings (e.g., ease of understanding, enjoyment in life) in a nonclinical lonely student group compared with those with SAD, i.e., > 70% in the student group vs 50% in the SAD group reported higher than somewhat in their satisfaction ratings. Specifically, those with SAD reported that they did not feel that +Connect helped them create new relationships or increase social confidence. While +Connect was not intended to create new relationships (rather the focus is on increasing the quality of existing relationships), it is important to consider modifying the app to assist young people with SAD because social interactions, including those suggested within the app, are likely to be significantly more difficult for them.

In the qualitative interview, at least four participants with SAD reported that they preferred actor videos over shared experience videos even though 87.5% of the group rated the shared experience videos as somewhat useful (see **Supplementary Table 4** for more details). All students found the shared experience videos more than somewhat useful and enjoyable. While previous studies that have found that content featuring people with lived experiences is highly acceptable to mental health consumers with similar experiences (49, 63), our findings also indicate that shared experience videos may also be useful for those without a mental illness, as long as the experience being portrayed is shared (in this case, loneliness). Expert videos (i.e., academics speaking to the audience) were the least enjoyable, which suggest that videos that provided either background or summary information should be relayed in a more youth-friendly format. Focus groups with young people with or without a mental disorder have already been engaged in the next iteration and have given feedback on how to relay seemingly useful but mundane information within animated videos instead of messages from video-recorded experts.

One strength of this study was the ability to triangulate qualitative and quantitative data to deepen our understanding of participants’ experiences using the app. For example, while satisfaction ratings for different video types would suggest that, overall, both participant groups preferred the shared experience videos, the in-depth interview data suggests that participants with SAD were split almost equally on their preference for shared experience or actor videos, while student participants were split almost equally between shared experience, actor, and expert videos. Further, their feedback provided a more nuanced understanding of the differences in video scores for “useful” versus “enjoyable.” Participants seemed to prefer actor videos based on the quality of the learning experience (i.e., usefulness), despite reporting them as less enjoyable. This suggests that useful content is more important to users than enjoyable content.

Uptake of the app was 91% and 87%, respectively, for students and those with SAD. While uptake was high for both groups, the SAD group had a higher attrition rate of 30.76% compared with half of that with those with no mental health disorders at 15.38%. However, for those who were retained, both groups completed more than 70% of the program. While the consumer-focused guidance within its development phase may have contributed to both acceptability and feasibility ratings, these findings suggest that clinicians and researchers have to think more deeply about how to engage those with SAD in digital interventions. Fortunately, +Connect was assessed to be safe for young people with or without SAD. While the study was underpowered to determine any meaningful difference pre-post, the evidence of a generally positive effect in quantitative ratings was consistent with qualitative data. No participant reported negative outcomes, and positive outcomes were driven by reflection, learning, and real-life application processes. In our exploratory analyses, we found that +Connect was more likely to benefit, or at least not cause any harm, to young people.

In order to ensure that feedback from this proof-of-concept trial can enhance the participant’s experience in future versions, we assessed its usability, and results indicated that participants found its format easy to navigate and the language easy to understand. Challenges presented in the app, however, may appear overwhelming for both groups, in that challenges were perceived as either time consuming or effortful. Furthermore, the current badge gamification system may require further revision, to encourage participation, with a participant with SAD suggesting changing reward schedules and a student noting that it was not motivating enough.

### Study Limitations and Future Directions

First, this study is statistically underpowered to determine the significant effects of pre-post intervention. However, our findings are supported by qualitative data that will assist in revising the app for use in a well-powered randomized control trial. There were several reasons related to the small *N*, which contributed to poor recruitment of young people with SAD: 1) there was change in management and clinician turnover at the recruitment site; 2) young people with SAD were particularly difficult to recruit, plausibly due to a high social avoidance of services.

Second, it is important to take into account that many research studies offer reimbursement to offset any potential costs the participant may incur during the study. In this case, participants were reimbursed to offset potential costs when logging into the app outside WIFI networks. While the completion of this program (84.66% to 90.26%) was high, it is unclear whether engagement with +Connect would be different in “real-world” settings.

Last, +Connect is merely a tool to deliver information to young people. A more tailored approach to assist young people to translate these skills to real life may be required for at least some young people. Such an approach may involve more clinician or peer interaction within safe and moderated chatrooms. Alternatively, the app might ask users for feedback about challenges, leading to a more titrated, multi-step approach for participants who rate challenges as unachievable for them. A more tailored approach may be especially helpful for young people who are lonely, with co-occurring mental ill health or clinical mental disorders. Feedback taken from this proof-of-concept app can be used to develop a second iterative version, with a focus on increasing therapeutic outcomes and improving engagement.

### Conclusion

Our proof-of-concept app +Connect was developed with an aim of addressing loneliness severity in young people. The development involved consumers aged 18 to 25 with or without a mental health disorder. In this pilot mixed methods study, we focused on pilot primary outcomes such as acceptability, feasibility, and safety; next, we explored the potential outcomes of the pilot. Our findings suggest that those with SAD may benefit from such interventions but may require more tailored support within the app in order to address attrition. Together with quantitative and qualitative data, there is a rationale to do further work such as modifying +Connect and examining its effectiveness within a pilot randomized controlled trial.

## Data Availability

The datasets generated for this study are available on request to the corresponding author.

## Author Contributions

All authors made a significant contribution to this manuscript. ML holds all chief investigator duties, including app development, study design, and write-up of this manuscript. TR contributed to the development of the app content, statistical analyses, and the write-up of this manuscript. RE was the postdoctoral research fellow in charge of the recruitment, contributed to the statistical analyses, and the write-up of this manuscript. KL contributed to the qualitative analysis. DP contributed to the development of the app content and the write-up of this manuscript. JG contributed to the development of the app content and the write-up of this manuscript.

## Funding

Funding was provided to ML *via* the Higher Education Research Participation Programs and Barbara Dicker Brain Sciences Foundation.

## Conflict of Interest Statement

The authors declare that the research was conducted in the absence of any commercial or financial relationships that could be construed as a potential conflict of interest.

## Footnotes

1 Actors in videos were under 25 years old at time of filming.

2 2Due to a technical issue with the survey, one participant did not contribute to the feasibility, acceptability, and usability ratings of the +Connect App.

3 Further app design feedback primarily focused on app design and navigation, additional content, additional notifications, increased login flexibility, gamification, and additional opportunities for social interaction. A list of participant improvement suggestions is provided in Supplementary Table 4.
