# Supplementary material for: A Pilot Digital Intervention Targeting Loneliness in Youth Mental Health
Source: Front Psychiatry. 2019 Aug 23;10:604. doi: 10.3389/fpsyt.2019.00604 (PMC6716464; doi:10.3389/fpsyt.2019.00604)
Supplement: Supplementary file 1 [file DataSheet_1.docx]

Supplementary Table 1: +Connect modules, aims, and tasks (1 of 2)

| Level | | Days | Module | Module Aims | Tasks |
| --- | --- | --- | --- | --- | --- |
| 1 | | 1 -2 | Introduction | Orienting the individual to objective of the app and normalizing loneliness as a human signal to connect | Tell Us About You! *^a, b^*  Why +Connect? *^a ,b, e^* |
| 2 | | 2 - 5 | Strengths^g^ | Helping individuals identify their own strengths and teach them ways to harness them when developing interpersonal relationships | Identify Your Strengths *^a, b, g^*  Using Your Strengths *^a,b,c^*  My Strengths *^a,b,d^* |
| 3 | | 6 -7 | Positive Emotions^g^ | Introducing a range of different positive emotions? Helping individuals to understand what elicits positive emotions for them | Positive Emotions *^a,b,c^*  What Makes You Feel Good? *^a,b,d^* |
| 4 | | 8 - 9 | Three Good Things^g^ | Introducing an additional exercise to elicit positive emotion.  Relaying that three good things exercise can be difficult at first, but with practice, it becomes easier. | Three Good Things *^a,b,c^*  Doing Three Good Things *^a,b,d^* |
| 5 | 10 - 12 | | Gratitude^g^ | Introducing an interpersonal focused gratitude exercise.  Emphasize the importance of going beyond to say thank you. Relaying that gratitude is difficult to do and can be confronting, however, doing the exercise can bring people closer. | Gratitude *^a,b, f^*  The Gratitude Exercise *^a,b,c^*  Showing Gratitude *^a,b,d^* |
| 6 | 13 - 15 | | Kindness^g^ | Introducing a non-confrontational way of opening up conversations with others. Relaying that acts of kindness does not have to cost the individual anything and small acts build up positive emotion for both the giver and receiver. | Kindness *^a,b, f^*  Showing Kindness *^a,b,c^*  Five Acts of Kindness *^a,b,d^* |
| 7 | 16 - 18 | | Balanced Relationships^g^ | Relaying the importance of reciprocal relationships and how it may be a balancing act. Acknowledging that when one is stress, it becomes more difficult to give back. | Balanced Relationships *^a,b, f^*  A Balancing Act *^a,b,c^*  Giving Back to Others *^a,b,d^* |
| 8 | 19 - 21 | | What is Savouring?^g^ | Encouraging the use of remembering positive memories shared with friends or loved ones. | What is Savouring? *^a,b, f^*  Savouring Memories *^a,b,c^*  Savouring Feels Good *^a,b,d^* |
| 9 | 22 | | Review Your Goals | Ensuring the participant refines their goals | Review Your Goals *^a,b^* |

*Note*. *^a^*refers to Mood Evaluation Log, *^b^* refers to Task, *^c^* refers to Actor video, *^d^* refers to Shared Experience Video, *^e^* refers to Expert Video, *^f^* refers to written content post and ^g^ refers to Challenge.

Supplementary Table 1: +Connect modules, aims, and tasks (2 of 2)

| Level | Days | Module | Module Aims | Tasks |
| --- | --- | --- | --- | --- |
| 10 | 23 - 24 | Sharing Positive News^g^ | Relaying the importance of sharing positive news but in a context (time, place, person) that makes sense. | Sharing Positive News *^a,b, f^*  Thinking Before Sharing *^a,b,c^* |
| 11 | 25 - 27 | Responding to Good News^g^ | Introducing four different ways in which one can respond to when a friend tells them positive news. Active constructive, passive constructive, active destructive, passive destructive responses are demonstrated. The impact on how one responds is demonstrated and the only response to build intimacy is active-constructive responses. | What Is An Active Constructive Response? *^a,b, f^*  Different Ways Of Responding *^a,b,c^*  Why Do Active Constructive? *^a,b,d^* |
| 12 | 28 - 29 | Self-disclosure | Relaying the importance of self-disclosure but in a context (time, place, person) that makes sense. | Self-disclosure *^a,b, f^*  How To Self-disclose *^a,b^* |
| 13 | 30 - 31 | Confidants^g^ | Relaying the importance of having a confidant in which one trust. The different kinds of people one can go to is encouraged. | What Is A Confidant? *^a,b^*  Learning To Confide *^a,b,c^* |
| 14 | 32 - 35 | Social Fears^g^ | To normalize social anxiety and to demonstrate that everyone has a level of social anxiety. The internal and external dialogues by people who are socially anxious can differ. | What Are Social Fears? *^a,b, f^*  Everyone Has Social Fears *^a, b, c^*  Nature Of Social Fears *^a,b,c^*  Overcoming Social Fears *^a,b,d^* |
| 15 | 36 - 39 | Close Relationships^g^ | To increase flexible thinking about how relationship closeness can change over time because of life events. Reconnecting with others may require forgiveness. Additionally, not trying so hard to be like others, can help facilitate the development of meaningful connections. | Changing Relationships *^a,b,c^*  Building Closeness *^a,b,d^*  Reconnecting With Others *^a,b,d^*  Being Yourself *^a,b,c^* |
| 16 | 40 - 42 | Wider Meaning^g^ and Goal Review | To encourage finding a life that is more meaningful and growing a sense of purpose (e.g., doing things for others). To encourage a positive outlook towards the future even though life hurdles are expected. | Wider Meaning *^a,b, f^*  Goals For The Future *^a,b^*  Looking To The Future *^a,b,e^* |

*Note*. *^a^*refers to Mood Evaluation Log, *^b^* refers to Task, *^c^* refers to Actor video, *^d^* refers to Shared Experience Video, *^e^* refers to Expert Video, *^f^* refers to written content post and ^g^ refers to Challenge.

Supplementary Table 2: Post-intervention acceptability ratings for +Connect modules for the student group

| Module | Not Helpful | | Somewhat Helpful | | Helpful | | Very Helpful | |
| --- | --- | --- | --- | --- | --- | --- | --- | --- |
|  | *n* | % | *n* | % | *n* | % | *n* | % |
| Gratitude | - | - | 2 | 18.18% | 7 | 63.63% | 2 | 18.18% |
| Three Good Things | - | - | 5 | 45.45% | 1 | 9.09% | 5 | 45.45% |
| What makes you feel good | - | - | 6 | 54.54% | 3 | 27.27% | 2 | 18.18% |
| Positive Emotions | 1 | 9.09% | 4 | 36.36% | 5 | 45.45% | 1 | 9.09% |
| Strengths^a^ | - | - | 6 | 54.54% | 2 | 18.18% | 3 | 27.27% |
| Goals | - | - | 8 | 72.72% | 3 | 27.27% | - | - |
| Kindness | 1 | 9.09% | 2 | 18.18% | 6 | 54.54% | 2 | 18.18% |
| Balanced Relationships | - | - | 4 | 36.36% | 4 | 36.36% | 3 | 27.27% |
| Savouring | 1 | 9.09% | 3 | 27.27% | 4 | 36.36% | 3 | 27.27% |
| Sharing Positive News | 1 | 9.09% | 2 | 18.18% | 5 | 45.45% | 3 | 27.27% |
| Active Constructive Responses | 1 | 9.09% | 3 | 27.27% | 5 | 45.45% | 2 | 18.18% |
| Self-Disclosure | - | - | 3 | 27.27% | 6 | 54.54% | 2 | 18.18% |
| Confidants | - | - | 4 | 36.36% | 3 | 27.27% | 4 | 36.36% |
| Social Fears | 1 | 9.09% | 2 | 18.18% | 4 | 36.36% | 2 | 18.18% |
| Changing Relationships^a^ | - | - | 3 | 33.33% | 4 | 44.44% | 2 | 22.22% |
| Building Close^a^ Relationships^a^ | - | - | 2 | 22.22% | 4 | 44.44% | 3 | 33.33% |
| Being yourself^a^ | 1 | 11.11% | 1 | 11.11% | 4 | 44.44% | 3 | 33.33% |
| Reconnecting with others^a^ | - | - | 3 | 33.33% | 3 | 33.33% | 2 | 22.22% |
| Wider Meaning^a^ | 1 | 11.11% | 3 | 33.33% | 3 | 33.33% | 2 | 22.22% |

*Note*. ^a^ two participants’ data points missing due to no reaching that stage of the +Connect program.

**Supplementary Table 3: Post-intervention acceptability ratings for +Connect modules for the social anxiety disorder group**

| Module | Not Helpful | | Somewhat Helpful | | Helpful | | Very Helpful | |
| --- | --- | --- | --- | --- | --- | --- | --- | --- |
|  | *n* | % | *n* | % | *n* | % | *n* | % |
| Gratitude | - |  | 3 | 37.5% | 4 | 50.00% | 1 | 12.50% |
| Three Good Things | - |  | 2 | 25.00% | 2 | 25.00% | 4 | 50.00% |
| What makes you feel good | - |  | 3 | 37.5% | 5 | 62.50% | - | - |
| Positive Emotions | - |  | 2 | 25.00% | 5 | 62.50% | 1 | 12.50% |
| Strengths^a^ | - |  | 3 | 37.5% | 5 | 62.50% | - | - |
| Goals | - |  | 2 | 25.00% | 6 | 75.00% | - | - |
| Kindness | - |  | 2 | 25.00% | 6 | 75.00% | - | - |
| Balanced Relationships | - |  | 4 | 50.00% | 3 | 37.5% | 1 | 12.50% |
| Savouring | 2 | 25.00% | 3 | 37.5% | 3 | 37.5% | - | - |
| Active Constructive Responses | - | - | 1 | 12.50% | 3 | 37.5% | 3 | 37.5% |
| Self-Disclosure | - | - | - | - | 5 | 62.50% | 3 | 37.5% |
| Confidants | - | - | 3 | 37.50% | 5 | 62.50% | - | - |
| Social Fears | - | - | 3 | 37.50% | 3 | 37.5% | 2 | 25.00% |
| Changing Relationships^a^ | - | - | 4 | 50.00% | 2 | 25.00% | 2 | 25.00% |
| Building Close^a^ Relationships^a^ | - | - | 2 | 25.00% | 5 | 62.50% | 1 | 12.50% |
| Being yourself^a^ | - | - | 5 | 62.50% | 3 | 37.5% | - | - |
| Reconnecting with others^a^ | - | - | 3 | 42.86% | 4 | 57.14% | - | - |
| Wider Meaning^a^ | - | - | 4 | 57.14% | 3 | 42.86% | - | - |

*Note*. ^a^ two participants’ data points missing due to not reaching that stage of the +Connect program.

Supplementary Table 4: +Connect video ratings for students and social anxiety group

|  | Student group | | | | | | Social anxiety group | | | | | |
| --- | --- | --- | --- | --- | --- | --- | --- | --- | --- | --- | --- | --- |
| Video Type | Not at all | | Somewhat | | Very much | | Not at all | | Somewhat | | Very much | |
|  | *n* | % | *n* | % | *n* | % | *n* | % | *n* | % | *n* | % |
| SEV were useful | - | - | 3 | 27.27% | 8 | 72.72% | 1 | 12.5% | 5 | 62.50% | 2 | 25.00% |
| SEV were enjoyable | - | - | 7 | 63.63% | 4 | 36.36% | 2 | 25.00% | 6 | 75.00% | - | - |
| EV were useful | - | - | 3 | 27.27% | 8 | 72.72% | 2 | 25.00% | 5 | 62.50% | 1 | 12.50% |
| EV were enjoyable | - | - | 3 | 27.27% | 8 | 72.72% | 4 | 50.00% | 4 | 50.00% | - | - |
| AV were useful | 2 | 18.18% | 4 | 36.36% | 5 | 45.45% | 2 | 25.00% | 5 | 62.50% | 1 | 12.50% |
| AV were enjoyable | 3 | 27.27% | 2 | 18.18% | 6 | 54.54% | 3 | 37.50% | 5 | 62.50% | - | - |

*Note.* SEV refers to Shared Experience Videos, ^b^EV refers to Expert Videos, AV refers to Actor Videos

Supplementary Table 5: Participant suggestions for +Connect refinement (1 of 3)

|  | SAD Group | | Student Group | |
| --- | --- | --- | --- | --- |
| Nature of feedback | *n* | Representative quote | *n* | Representative quote |
| *Improved navigation/ design* | 5 | “I wish there was the ability to look back over videos and re-watch them.”  “on the home page maybe have…something to remind you of your strengths and a progress thing for your goals.”  “Subtitles for videos would be neat for scenarios when I don't have earphones in public” | 8 | “On the mood board they have the sliders between 0 and 100. Yeah I found that irritating to use.... I would prefer if it said maybe like the buttons of like somewhat disagree and like strongly agree.”  “if [the challenges] all had their own individual theme to them; their own individual structure.”  “I think part of the reason three good things was good because it was very easy to remember. It’s got a very ear-wormy name. So if you can come up with like really three-word or easy to remember names for the different activities, it might be more powerful.”  “Most of the challenges required you to write something down at the start and write something down at the end. But you couldn’t write something down at the start because if you write something down it thinks that you’ve completed the challenge”  “You can add animations in it, you can make it more colourful, definitely” |

Supplementary Table 5: Participant suggestions for +Connect refinement (2 of 3)

|  | SAD Group | | Student Group | |
| --- | --- | --- | --- | --- |
| Nature of feedback | *n* | Representative quote | *n* | Representative quote |
| *Additional content* | 3 | “maybe even just strategies to help with like mental health issues.”  “some suggestions in the app for reaching out to services that are already there? Like eheadspace or Men’s Health Line,”  “a bit more like… ‘research tells us’” | 6 | “[the videos] should also focus a little more on like mental health”  “more insight about how to go about building relationships with people. It should have focussed more on friendships.”  “it would be interesting to maybe add some psychological findings or studies or aspects that are kind of contradictory, are more on an enhanced level.”  “I think more real life examples of people overcoming a certain situation.”  “Like a comic or anything and you have to pick the answers the person should give…. For example, at a party and then you see an avatar coming and saying anything and you can choose between three different things to say and then the avatar reacts depending on what you say and then the story develops in a different way.”  “more of the challenges.”  “Rather than 2 to 3 questions, 5, 6 questions.” |
| *More notifications* | 5 | “More notification options. For challenges have a more regular reminders.” | 2 | “if the challenge was 7 days long, it would be good to like have a daily reminder.”  “every time a mood log needed to be updated, a notification, and like a second notification.” |

Supplementary Table 5: Participant suggestions for +Connect refinement (3 of 3)

|  | SAD Group | | Student Group | |
| --- | --- | --- | --- | --- |
| Nature of feedback | *n* | Representative quote | *n* | Representative quote |
| *Increased flexibility of login* | 3 | “There was one thing that I didn’t like. There was the fact that you couldn’t do more than one module in a day… if I’d missed a couple of days I wanted the ability to kind of catch up” | 3 | “If I suppose I miss out on this day, the next day whatever is pending I should be able to do. I don't think that's happening, I'll have to wait another 24 hours.”  “Maybe have an app like on the computer itself as well. Because not everyone uses their phone”  “I didn’t really like the time limit [on challenges].” |
| *Increased gamification with different rewards* | 3 | “bonus points or something for completing [challenges] in the time frame” | 2 | “[for the challenges] some type of reward. Because I felt I didn’t really need to do them” |
| *More opportunities for social interaction* | 2 | “Maybe like a chat forum or something. Kind of interacting with other participants… Like get some sort of support network going. Maybe like do a, challenge a friend to do it with you. And have a basis of discussion. Like I watched this video, what did you think of it? I watched this video.” | 2 | “even small little things like teamwork challenges or like little challenges where you need someone else to interact with you or help you out to do that.” |
